# Layer-by-Layer Nanoparticles of Tamoxifen and Resveratrol for Dual Drug Delivery System and Potential Triple-Negative Breast Cancer Treatment

**DOI:** 10.3390/pharmaceutics13071098

**Published:** 2021-07-20

**Authors:** Ali A. Al-jubori, Ghassan M. Sulaiman, Amer T. Tawfeeq, Hamdoon A. Mohammed, Riaz A. Khan, Salman A. A. Mohammed

**Affiliations:** 1 Division of Biotechnology, Department of Applied Sciences, University of Technology, Baghdad 10066, Iraq; ali.adnan.aljubori@gmail.com (A.A.A.-j.); ghassan.m.sulaiman@uotechnology.edu.iq (G.M.S.); 2 Molecular Biology Department, Iraqi Center for Cancer and Medical Genetics Research, Mustansiriyah University, Baghdad 10052, Iraq; amer.tawfeeq@iccmgr.org; 3 Department of Medicinal Chemistry and Pharmacognosy, College of Pharmacy, Qassim University, Qassim 51452, Saudi Arabia; ham.mohammed@qu.edu.sa (H.A.M.); ri.khan@qu.edu.sa (R.A.K.); 4 Department of Pharmacognosy, Faculty of Pharmacy, Al-Azhar University, Cairo 11371, Egypt; 5 Department of Pharmacology and Toxicology, College of Pharmacy, Qassim University, Qassim 51452, Saudi Arabia

**Keywords:** tamoxifen, resveratrol, layer-by-layer methods, drug delivery, cellular uptake, biocompatibility, cytotoxicity, P53 and caspase-8 pathway, MCF-7 cells, CAL-51 cells

## Abstract

Nanoparticle development demonstrates use in various physicochemical, biological, and functional properties for biomedical applications, including anti-cancer applications. In the current study, a cancer therapeutic conjugate was produced consisting of tamoxifen (TAM) and resveratrol (RES) by layer-by-layer (LbL) nanoparticles based on lipid-based drug delivery systems and liquid crystalline nanoparticles (LCNPs) coated with multiple layers of positively charged chitosan and negatively charged hyaluronic acid for the evaluation of biocompatibility and therapeutic properties against cancer cells. Multiple techniques characterized the synthesis of TAM/RES–LbL-LCNPs, such as Fourier-transform infrared spectroscopy (FTIR), X-ray crystallography (XRD), Zeta potential analysis, particle size analysis, Field Emission Scanning Electron Microscope (FESEM), and Transmission electron microscopy (TEM). The *in vitro* cytotoxic effects of TAM/RES–LbL-LCNPs were investigated against human breast cancer cell line, Michigan Cancer Foundation-7 (MCF-7), and human triple-negative breast cancer cell line, Centre Antoine Lacassagne-51 (CAL-51), using various parameters. The 3-(4,5-dimethylthiazol-2-yl)-2,5-diphenyltetrazolium bromide (MTT) assay confirmed that the treatment of cells with TAM/RES–LbL-LCNPs caused a reduction in cell proliferation, and no such inhibition was observed with human normal liver cell line: American Type Culture Collection Cell Line-48 (WRL-68 [ATCC CL-48]). Fluorescent microscopy examined the ability of Fluorescein isothiocyanate (FITC) to bind to TAM/RES–LbL-LCNPs along with their cellular uptake. Apoptosis determination was performed using hematoxylin–eosin and acridine orange–propidium iodide double staining. The expression of P53 and caspase-8 was analyzed by flow cytometry analysis. An *in vivo* study determined the toxicity of TAM/RES–LbL-LCNPs in mice and assessed the functional marker changes in the liver and kidneys. No significant statistical differences were found for the tested indicators. TAM/RES–LbL-LCNP treatment showed no apparent damages or histopathological abnormalities in the heart, lung, liver, spleen, and kidney histological images. The current findings observed for the first time propose that TAM/RES–LbL-LCNPs provide a new and safer method to use phytochemicals in combinatorial therapy and provide a novel treatment approach against breast cancers.

## 1. Introduction

Cancer is a foremost contributor to mortality, with a documented one in seven deaths globally. The global cancer burden is predicted to be 21.7 million new cases and 13 million deaths by 2030 [1]. According to a 2018 report in Iraq, of the 12 types of prevalent cancer, lung and breast cancers had a prevalence rate of 20.5% and 17.4%, followed by 15.4% and 6.8% for bladder and prostate cancer, respectively [2]. Breast cancer remains a significant contributor to female morbidity and mortality [3]. According to the American Cancer Society, as of 2019, global statistics estimated new breast cancer diagnosis cases of 268,600 and 2670 among women and men, respectively, with approximate mortality of 41,760 women and 5000 men in the same year [4]. Mortality is alarming due to insufficient resources for prevention, diagnostics, and therapy of breast cancer. In women, hormone-dependent estrogen receptor (positive ER) breast cancer contributes to 75% of overall breast cancer cases. This observation has drawn the attention of research toward developing effective drugs to treat hormone-dependent breast cancer. Furthermore, estrogen, a female sex hormone, demonstrated an essential part in the induction and advancement of breast malignancy. Hence, ER remains a crucial focus for estrogen hormone-dependent breast cancer treatment [4].

Triple-negative breast cancer (TNBC) involves loss of expression of ER, the progesterone receptor (PR), and the human epidermal growth factor receptor-2 (HER2). As a result, TNBC demonstrates the worst prognosis with limited therapeutic options [5], more so due to these receptors commonly used in targeting tumor cells, making it extremely difficult to target TNB cancer cells [6,7]. Systemic chemotherapies can be applied towards TNB cancer therapy [8], but a lack of specific targeting leads to significant healthy tissue damage [9]. Chemotherapy employs common therapeutic drugs with a 20% response rate achievement but it also involves increased chemical resistance, systemic toxicity, and significant collateral damage leading to myelosuppression, immunosuppression, cardiotoxicity, neuropathy, and myalgia [10]. Unfortunately, even these treatment problems also lead to clinical loss, making TNBC the worst average result for women among all the breast cancer subtypes due to the aggressive nature of the tumor, slow diagnosis, and early-stage non-specific symptoms. It is therefore vital to establish new clinical approaches towards TNBC treatment [11]. The discovery of specific TNBC biomarkers for diagnosing and curative uses is a crucial clinical pre-requisite. Nanotechnology provides the desired platform to find less expensive biomarkers [12].

Currently, there is a tendency to look for natural ingredients from plants demonstrating therapeutic benefits to human health with interesting biological properties supporting pharmacological therapy [13]. Resveratrol (RES), a non-flavonoid polyphenol natural bioactive compound found in various plants [14], demonstrated cancer chemo-preventive properties in 1997 [15]. However, even though RES was demonstrated to be a promising anti-cancer candidate in several *in vitro* cancer studies, it was restricted for clinical use due to its quick elimination from the body, and because it lacked therapeutically relevant levels in the bloodstream [16]. Nevertheless, the combination of RES with other natural agents demonstrated its usefulness in advanced cancer stages through several routes, thereby inhibiting tumor development and oncogenic signaling [17].

In nanoscience, nanoparticles (NPs) have various physiochemical, biological, and functional characteristics in the biomedical field. The ideal nanoparticle size (1–200 nm) and conformation define the trajectory dynamics of NPs that are decisive for the formulation of nanomedicine. In addition, the surface charge and drug encapsulation ability of NPs are essential factors for the accurate selective delivery of drugs using a unique conjugate ligand against the target receptor for cancer cells. Furthermore, other properties such as increased drug loading ability, longer half-life with mostly minor systemic toxicity, increased internalization into the tumor *via* endocytosis, sustained and regulated release of cytotoxic drug over the suitable duration and time along with body excretion are significant for NPs as theranostics in cancer therapeutics [18].

Tamoxifen citrate (TAM) is commonly employed as a non-steroidal estrogen antagonist in ER+ breast cancer therapeutics, in addition to long-term preventive use in risky and post-menopausal women [19]. TAM acts by causing apoptosis through cell-cycle arrest [20]; it also acts as an anti-oxidant [21] and may stimulate tumoricidal effects on ER-cells through increased inhibitory growth factor production [22]. Although promising, TAM is eliminated *via* hepatic first-pass metabolism with a detectable drug concentration up to 24 h of oral administration [23].

To enhance the anti-cancer efficiency, we produced a cancer therapeutic conjugate involving RES and TAM by layer-by-layer nanoparticles (LbL) based on lipid-based drug delivery systems (LbDDS) and liquid crystalline nanoparticles (LCNPs) to evaluate their potential therapeutic properties against cancer cells. LbL provides a flexible approach in coating water-soluble as well as insoluble drugs, with its limitations during the preparatory process being that it is tedious and time-consuming and wastes materials [24]. A wide range of synthetic and biological polymeric materials can be coated on the substrate, such as hyaluronic acid (HA), chitosan (CS), poly(ethylene glycol) (PEG), and others [25]. The natural polysaccharide-based polyelectrolytes, negatively charged HA, and positively charged CS demonstrate exceptional antibacterial properties, biocompatibility, and biodegradability through the combination with oppositely charged polymers into nano-complexes or modification of NPs [26]. Additionally, HA’s specificity in binding to CD44 receptors, overexpressed in tumor cells, has been used as an active targeting moiety [27] in addition to its use in joint lubrication, tissue hydration, wound healing, and as a moisturizer in cosmetic and pharmaceutical applications. CS is used in surgical sutures and drug-controlled release materials [28]. CS and HA were recently coated on LbL to produce pH-responsive polymer films and were used in bacterial [29] and cancer treatment [28,30]. LCNPs provide fluidic and particulate delivery system advantages in addition to structure flexibility, increased colloidal strength, and prolonged-release profile [31] to amphiphilic, hydrophilic, and hydrophobic drug forms, even in unfavorable environments [32]. Several anti-cancer drugs have been used for LbL encapsulation, including TAM, paclitaxel, camptothecin, prednisolone, and RES [25], in addition to proteins [33], peptides, and nucleic acids such as RNAi [34], DNA, and oligonucleotide sequences estimating immunogenicity [35], gene editing or transcription [36]. No study has been investigated on TAM/RES-loaded LCNPs along with their therapeutic potential. However, resveratrol is known to have anti-cancer properties in addition to its role in diabetes and neurodegenerative diseases. RES orally is limited due to its poor water solubility, stability, and pharmacokinetics profile. At the same time, LbL-loaded RES circumvented these issues and has a promising preventive and therapeutic use [37]. Recently the Santo group, using an in vivo model, demonstrated 1.7 times significant systemic exposure of RES–LbL NPs compared to the free RES in addition to 24 h stability in gastric and intestinal conditions [38]. Additionally, cholesterol-conjugated bovine serum album (BSA)-TAX NPs demonstrated significant anti-cancer activity in the 4T1 cancer cell line than BSA NPs and BSA-TAX NPs [39]. LbL is a promising approach in anti-cancer therapy; even though it is in its early application phase, it can be fast-tracked by finding more biocompatible materials and developing more biocompatible techniques, as biosafety is a critical factor in transiting to the clinical side [24]. To the best of our knowledge, this is the first report on the anti-cancer effects of TAM/RES-loaded LCNPs on human breast cancer cells. The impact against the human breast cancer cell line MCF-7 and human TNBC cell line Cal-51 was evaluated. In addition, efficiency and biocompatibility to the normal human liver cell line (WRL-68) and human red blood cells were tested for this system. An in vivo study was conducted to determine TAM/RES–LbL-LCNP toxicity in mice, and functional marker changes in the liver and kidneys were assessed.

## 2. Materials and Methods

### 2.1. Materials and Reagents

Tamoxifen citrate (TAM), resveratrol (RES), chitosan (CS; low molecular weight, 85% acetylation, viscosity, 20–200 cps), hyaluronic acid (HA) (MW, 100 kDa), and poloxamer 407 (P407) were purchased from Sigma Chemical Co. (St. Louis, MO, USA) with purity ~98%. Acridine orange, ethidium bromide (AO/EtBr), trypsin-EDTA, dimethyl sulfoxide (DMSO), 3-(4,5-dimethylthiazal-z-yl)-2,5-diphenyltetrazolium (MTT), fluorescein isothiocyanate (FITC), crystal violate stain, fetal bovine serum (FBS), and glyceryl monooleate (MO) were purchased from Sigma Chemical Co. (St. Louis, MO, USA). Roswell Park Memorial Institute (RPMI)-1640 was ordered from Euro Clone (Milan, Italy). Fluorescein p53 and caspase-8 staining package were from Thermo Fisher Scientific (Waltham, MA, USA). Antibiotics such as penicillin and streptomycin were provided from Biosource International (Nivelles, Belgium). The remaining chemicals and reagents were applied with analytical grades.

### 2.2. Cell Lines Cultures

The human breast cancer cell line, Michigan Cancer Foundation-7 (MCF-7), human TNBC cell line, Centre Antoine Lacassagne-51 (CAL-51), and normal human liver cell line, American Type Culture Collection Cell Line-48 (WRL-68 [ATCC CL-48]) were supplied by the Iraqi center for cancer and medical genetic researches (ICCMGR), Mustansiriyah University, Baghdad, Iraq. RPMI-1640 supplemented with 10% FBS, 2 mM L-glutamine, and 20 mM HEPES was employed to culture MCF-7 cells using tissue culture treated flasks (T 25 cm^2^; Corning, AZ, USA) under the conditions of 5% CO_2_ and 37 °C. WRL-68 cells were maintained under similar conditions as those used for cancer cell lines.

### 2.3. Preparation of TAM/RES Loaded LCNPs

TAM/RES-LCNPs were formulated using the hydrotrope method with minor modification. Briefly, TAM (50 mg) and RES (50 mg) were suspended in ethanol (4 mL) followed by mixing MO (500 mg) in ethanol (0.44 mL). The resulting mixture was introduced dropwise onto 20 mL of Poloxamer-407 solution (500 mg with 100 mL of distilled water (DW) with respect to the total dispersion volume). The mixture was maintained under continuous stirring at 50 rpm for 12 h to evaporate the ethanol. The resultant TAM/RES-LCNPs were subjected to homogenization by vortex, sonication for 10 min and water bath 40 °C. After that, the system was left for equilibrium for 24 h [40].

To prepare the LbL-coated TAM/RES–LCNP, negatively charged TAM/RES-LCNPs (1 mL) were measured with a cationic CS solution (1 mg mL^−1^; pH 4.5) at volume (0.5 mL) followed by 30 min incubation with mild stirring (100 rpm). Finally, a second negatively charged layer was added by titrating 0.25 mL of anionic HA solution (3 mg mL^−1^; pH 7) against 1 mL of CS-coated TAM/RES-LCNPs under mild stirring (100 rpm) for 30 min [40].

### 2.4. Release Profile of TAM/RES–LbL-LCNPs

The release of TAM and RES loaded on LCNPs in buffers of different pH values was established by applying the previously published method [41]. Briefly, 5 mg of TAM, RES, and TAM/RES–LbL-LCNPs were resolved in 5 mL Phosphate Buffered Saline (PBS) of either physiological pH of 7.4 or acidic pH of 5.0 and incubated at room temperature (RT) for different time points (0.5, 1, 1.5, 2, 3, 4, 6, and 12 h). At each treatment, the absorbance measurements were recorded by the UV–vis spectrophotometer for TAM and RES. The concentrations of the released TAM and RES were calculated using the following formula and standard curve calibration:

(1) $$Camulative\;Release\;\%=\left(Wr/Wt\right)\times 100$$

where *Wr* is the TAM or RES weight in each release, and *Wt* is the TAM or RES theoretical load weight.

### 2.5. Characterization of TAM/RES–LbL-LCNPs

The TAM or RES and TAM/RES–LbL-LCNPs were visualized with a UV–vis spectrum using a Shimadzu Europe UV-1650PC Spectrophotometer (Tokyo, Japan). UV–vis spectroscopic measurement was done with continuous scanning (200–900 nm). X-ray diffractometer (XRD-6000, ADX-2700 USA) characterized prepared NPs crystals (30 mA current; 40 kV voltage). A Cu Kα incident beam (λ = 1.542 Å) at 2θ = 5°–40° identified particles’ diffraction patterns. Fourier-transform infrared spectroscopy (FTIR), was conducted using 8400S (Tensor 27, Netherlands) (4000–400 cm^−1^ spectral range, 4 cm^−1^ resolution) with an attenuated total reflection mode. A field emission scanning electron microscope (FESEM) using a MIRA 3 TESCAN (Brno, Czech Republic) was used for identification of the synthesized conjugate’s morphological features. A transmission electron microscope (TEM, Zwiss, Germany) operating at 400 kV was used to identify the morphological features and conjugate distribution.

### 2.6. Sampling and Preparation of Human Blood

Fresh blood from human donors was sampled into heparin-coated tubes using the earlier described method [42], according to the National Institute of Health and Food and Drug Administration and Helsinki’s declaration and regulation as a statement of ethical principles. Permissions were obtained from the hospitals of the medical city in Baghdad, Iraq, and approved by the institutional ethical committee of the University of Technology, Baghdad, Iraq (Ref. No. AS 2411/22/10/2019).

### 2.7. Blood Compatibility Assay

The whole blood was collected (200 mL) and mixed with PBS (1600 mL), and the mixture was used with each of the six concentrations of TAM, RES, and TAM/RES–LbL-LCNPs (200 mL). DDW was employed as a positive control that ensures 100% hemolysis, while PBS was used as a negative control with 0% hemolysis. Triplicate samples were incubated (37 °C; 1 h). Following centrifugation (700 rpm; 5 min), a reading of the absorbance was carried out using a UV–vis spectrophotometer (541 nm) [43]. The formula below was applied to determine the hemolysis percentage:

(2) $$\mathrm{Hemolysis}\;\%=\left(\frac{OD_{s}-OD_{n}\;}{OD_{p}-OD_{n}}\right)\times 100$$

where *OD*_s_ is a sample under test, *OD*_n_ and *OD*_p_ are negative and positive controls, respectively.

### 2.8. Cytotoxicity against Cell Lines

Flat-bottom culture plates (96-well; Falcon, USA) were used to prepare a seeding of cancer cell line suspension (200 μL, 1 × 10^5^ cells mL^−1^). First, cells were grown in the exponential growth phase (48 h) followed by 24 h treatment with the tested concentrations of TAM/RES-LCNPs. Secondly, cells were labeled with MTT in PBS (100 µL; 10–15 min; 37 °C). Next, the stain was washed using tap water, and air bubbles were dissolved using DMSO (50 µL; 10 min). Cell cultures on a microplate reader (ELx 800, Bio-Tek Instruments Inc., Winooski, VT, USA) were measured at 492 nm [44]. The formula below was applied to determine the inhibition percentage:

(3) $$\mathrm{Inhibition}\;\mathrm{rate}\;\left(\%\right)=\left(\mathrm{Abc}\;–\;\mathrm{Abs}/\mathrm{Abc}\right)\times 100$$

where Abc and Abs are control and tested samples’ OD, respectively.

### 2.9. Clonogenicity Survival Assay

The assay was used to analyze colony formation’s inhibitory levels by MCF-7 or Cal-51 cells as described by Sulaiman [45]. Briefly, the cells were plated in 24 well plates (density of 800 cells mL^−1^) for 24 h followed by treatment with IC_50_ concentrations of TAM/RES–LbL-LCNPs. Next, the media of the confluent monolayer cell was discarded and washed with PBS. Finally, fixation and staining steps with crystal violet were conducted, followed by washing to remove excess dye from the samples and photographed.

### 2.10. Cellular Uptake of TAM/RES–LbL-LCNPs

Fluorescent-labeled TAM/RES–LbL-LCNPs and the conjugate were obtained according to the previous method [46]. Briefly, a mixture was prepared involving FTIC stain (1 μg mL^−1^) and TAM/RES–LbL-LCNPs and incubated 24 h at RT. Next MCF-7 cells were incubated (density of 1 × 10^5^ cells mL^−1^) for 24 h followed by the addition of test concentrations of FITC- TAM/RES–LbL-LCNPs and incubation for another 24 h. Then, cells were washed with PBS and fixed with 5% glutaraldehyde followed by nuclei staining and images obtained with a fluorescence microscope.

### 2.11. Acridine Orange–Ethidium Bromide (AO/EtBr) Dual Staining

The AO/EtBr dual staining method was applied for the induction of MCF-7 or CAL-51 cell death, according to the method by Sulaiman with a slight modification [45]. Briefly, cells were seeded (density 1 × 10^5^ cells per mL) using RPMI in 96 well microtiter plates and incubated overnight. IC_50_ concentrations of TAM/RES–LbL-LCNPs were then applied for 24 h followed by washing with PBS, applying dual fluorescent dyes (100 μL) to the cells, and finally visualized under a fluorescent microscope.

### 2.12. Hematoxylin and Eosin Staining

The MCF-7 or CAL-51 cells were prepared for morphological examinations. The cells were grown in T-50 culture flasks (density of 1 × 10^5^ cells mL^−1^) and incubated for 48 h. Further, the cells were treated with the IC_50_ value dose of TAM/RES–LbL-LCNPs after 24 h. Next, the media were removed, and the cells were fixed with formalin (10%) for 3–5 min and washed with DW three times. Next, the cells were dehydrated with increasing ethanol concentrations (70, 90, and 100%, 2 min each), washed twice with DW for 2–5 min, stained with hematoxylin, and again washed for 1 min with DW. Next, the cells were stained with eosin for 10–15 min and washed with DW. Finally, the cells were treated with 100% ethanol, followed by glycerin, and were examined under an inverted microscope at 20× magnification [47].

### 2.13. Flow Cytometry Assay

The p53 and caspase-8 activation states were determined with the fluorescein p53 and caspase-8 staining method. Briefly, the MCF-7 or CAL-51 cell line (4 × 10^4^ cells mL^−1^) was cultured in 5 mL of medium and incubated at 37 °C for 24 h. Cells were then exposed to each compound (10 μg mL^−1^) for 24 h. After incubation, the treated cells were extracted and washed 2 times with 1 × ice-cold PBS. The pellets were extracted, and the cell density (1 × 10^6^ cells mL^−1^) was reset using the growth medium. The prepared cells were then incubated with FITC-IETD-FMK (1 mL) at 37 °C for 60 min, which was used to identify caspase-8 and FITC-anti-p53. After the incubation time, the cells were washed 2 times with a buffer wash (0.5 mL). The stained cells were then exposed to flow cytometry. Data was measured and evaluated using the BD Accuri C6 software [48].

### 2.14. In Vivo Assays

#### 2.14.1. Laboratory Mice

Male mice four to five weeks old, naïve BALB/c (n = 15) weighing 19–21 gm, obtained from the animal house facility by Iraqi Center for Cancer and Medical Genetic Research, Mustansiriyah University, Baghdad, Iraq. The animals were maintained in individual polyacrylic cages housing five mice per cage with a chow diet and water ad libitum seven days before the beginning of the experiments. The animals were maintained at RT (24 ± 2 °C) and relative humidity of 55 ± 10% with a controlled light-dark cycle of 12:12 h. The institutional Research Ethics Committee approved the experimental procedure and animal care (Approval ID-2019-UT-3 by Animal Care and Ethics Committee at Biotechnology Division, Applied Sciences Department, University of Technology, Baghdad, Iraq) as per the Guidelines of the U.S. National Institutes of Health (NIH) Guide for the Care and Use of Laboratory Animals (NIH Publication No. 86–23, revised in 1996).

#### 2.14.2. Toxicity Assay

Fifteen mice were randomly divided into three groups, one control group, and two experimental groups, with two doses of TAM/RES–LbL-LCNPs (25 mg kg^−1^ or 150 mg kg^−1^). Mice were intraperitoneally (i.p.) injected with 100 µL of TAM/RES–LbL-LCNPs (Figure S1) every second day, where the final volume was adjusted according to each mouse weight. The control group received saline. Records of body weight and behavior were taken before the treatment during the experiment. Mice were sacrificed from each group following treatment on day 7 and day 14 (Figure S2). The liver, spleen, heart, kidney, and lungs (Figure S3) were excised immediately, and blood was obtained using cardiac puncture and centrifuged (3000 rpm; 10 min) for serum collections. PBS was used to wash selected organs, fixed with 10% formalin, and dispensed in a paraffin module EG 1150H (Leica, Germany). After paraffin embedding, tissues were sectioned (microtome RM2255, Leica, Germany) and stained with hematoxylin and eosin (H&E). The sera samples were collected and kept at −20 °C to estimate liver function enzymes, AST, ALP, and ALT, using the colorimetric method. Additionally, to evaluate the kidney functions, blood urea, serum creatinine, and uric acid were estimated using the enzymatic method [49,50].

### 2.15. Statistical Analysis

The significance of variances among groups was examined by applying the one-way analysis of variance (ANOVA, Tukey Test), statistically significant differences being set at * *p* ≤ 0.05 or ** *p* ≤ 0.01. Data were presented as mean ± standard deviation, while statistical significances were determined by GraphPad Prism version 6 (GraphPad Software Inc., La Jolla, CA, USA) [51].

## 3. Results and Discussion

### 3.1. Preparation of TAM/RES–LbL-LCNPs

**Figure 1:**
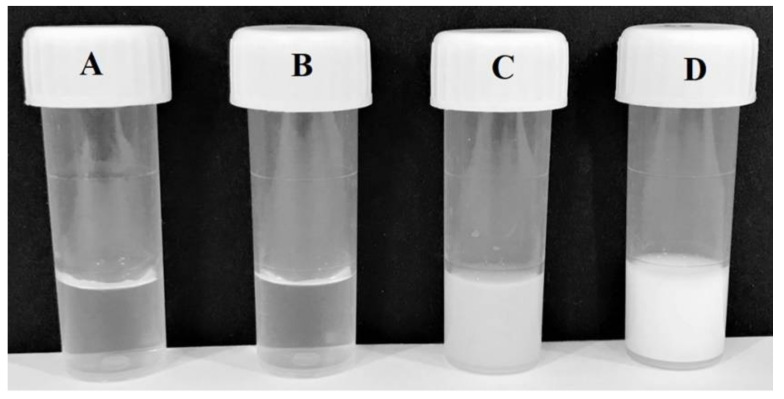
Image showing chemical reaction steps for tamoxifen (TAM)- and resveratrol (RES)-loaded liquid crystalline nanoparticles (LCNPs) and layer-by-layer (LbL) nanoparticle-coated TAM/RES-LCNPs. The color change was observed within the reaction: (**A**) TAM, (**B**) RES, (**C**) preparation step 1, (**D**) preparation step 2.

Figure 1 represents the preparation of LbL-coated LCNPs of TAM and RES. As seen in the figure, TAM and RES-LCNPs was formulated first using dilution and by increasing the solubility of water-insoluble lipids of monoolein through the hydrotrope method. After that, a CS layer followed by an outer HA layer were self-assembled on the proposed TAM and RES-LCNPs. The magnified part shows the liquid crystalline phase constituting hydrophobic lipid domains containing TAM and RES molecules and the hydrophilic water channels. The multilayer coating depends on the electrostatic interactions of two oppositely charged polyelectrolytes; thus, the present study aimed to maximize both HA and CS charge density, mainly depending on pH [40]. For this purpose, HA solution (pH 7) ensured full carboxylate group ionization, and CS was dissolved in acetate buffer (pH 4.5) to achieve maximum protonation of amino groups [40].

### 3.2. In Vitro Release of TAM/RES–LbL-LCNPs

**Figure 2:**
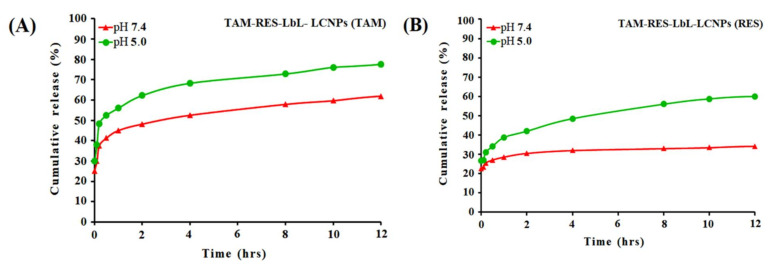
In vitro release profile of TAM–LbL-LCNPs (**A**) and RES–LbL-LCNPs (**B**) in PBS at pH 7.4 and pH 5.0.

The release kinetics of TAM and RES in physiological (pH 7.4) and acidic (pH 5.0) conditions were employed using UV–visible spectroscopy (Figure 2). The results demonstrated a time-correlated increase in TAM in acidic pH for up to 1 to 2 h. (~65%), which reached saturation (~80%) at 12 h, while the drug release was followed up with RES up to 1 to 2 h (~42%), which reached saturation (~57%) at 12 h. In physiological pH, in TAM and RES, a lower release (~55 and 30%, respectively) was observed than in acidic pH. It is well known that the physiological pH level is about 7.4, whereas pH at the tumor microenvironment is acidic (~5.0). The results revealed the ability of TAM/RES–LbL-LCNPs to accelerate TAM and RES delivery to the cancer cells [52]. It also confirms the long-term delivery of drugs from the nanosphere. Moreover, TAM and RES were released from the nanosphere at a similar rate, which can improve the bioavailability of TAM and decrease some side effects of TAM. A possible explanation may be that a more polyelectrolyte complex due to the increased mass ratio for CS-HA leads to the increasing amount of formed TAM/RES–LbL-LCNPs, resulting in the increased encapsulation efficiency and loading capacity in TAM/RES–LbL-LCNPs [53].

### 3.3. UV–Vis Spectrum Analysis

**Figure 3:**
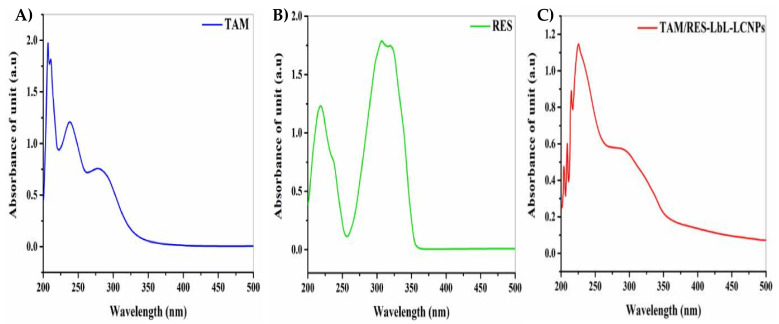
UV–vis spectroscopy analysis of (**A**) TAM, (**B**) RES, and (**C**) TAM/RES–LbL-LCNPs.

The data confirmed the formation of TAM/RES–LbL-LCNPs and the conjugation of both TAM and RES. As seen in Figure 3, the absorption spectrum of TAM and RES was seen at 207 and 308 nm, respectively (Figure 3A,B). Furthermore, the wavelength at maximum absorbance of TAM/RES–LbL-LCNPs demonstrated considerable shifting from the unloaded TAM and RES to be at λmax ~226 nm and λmax ~292 nm (Figure 3C), confirming that the feasibility of combining drug molecules and their ability to alter photo–physical properties. The preparation of TAM/RES–LbL-LCNPs was achieved following a chemical method, and the change in their UV–visible spectrum was observed.

### 3.4. X-ray Diffraction Characterization

**Figure 4:**
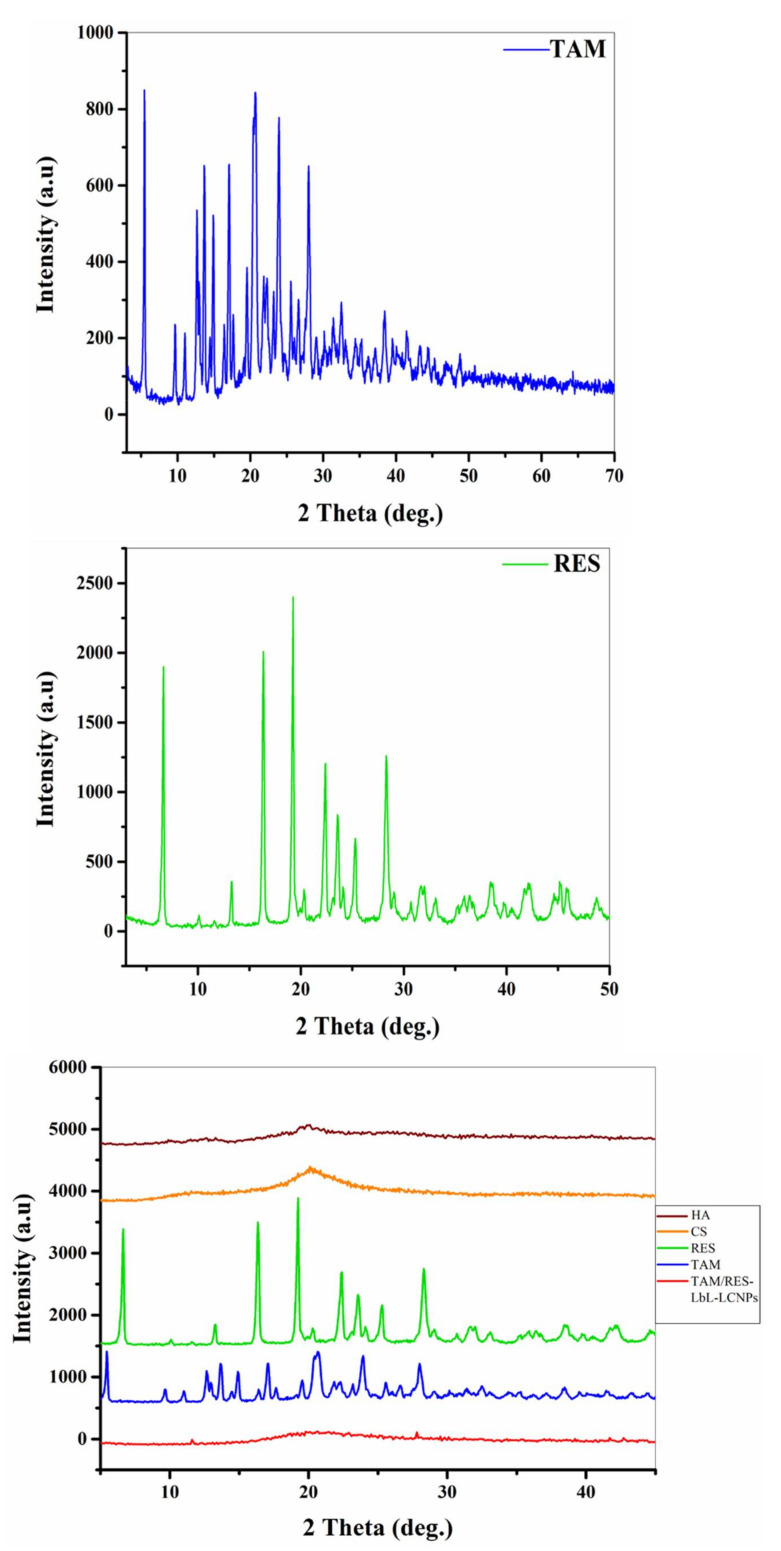
XRD spectrum patterns of TAM, RES, and other TAM/RES–LbL-LCNP preparation components. HA: hyaluronic acid; CS: chitosan; RES: resveratrol; TAM: tamoxifen citrate.

The structures of TAM, RES, and TAM/RES–LbL-LCNPs were demonstrated by XRD, as seen in Figure 4. The diffraction peaks of TAM were observed at 5.56°, 12.57°, 13.61°, 14.91°, 16.99°, 20.46°, 23.88°, and 28°, while RES displayed characteristic peaks of 2θ of 6.62°, 13.27°, 16.28°, 19.28°, 22.39°, 23.57°, 25.40°, and 28.32°, indicating their high crystalline structure. On the other hand, TAM/RES–LbL-LCNPs showed fewer but distinct peak intensities, indicating that TAM/RES–LbL-LCNPs were either molecularly dispersed in MO or an amorphous state in the matrix. The decline in crystallinity and reduced particle size could result in maximum entrapment efficiency, solubility, and bioavailability of TAM/RES–LbL-LCNPs. According to Scherrer’s formula, larger full width at half maximum (FWHM) values indicate smaller particle sizes [54]. However, the FWHM values of the TAM/RES–LbL-LCNP peaks were higher than those observed in TAM or RES (data not presented).

### 3.5. Fourier Transform Infrared Spectroscopy (FTIR) Analysis

**Figure 5:**
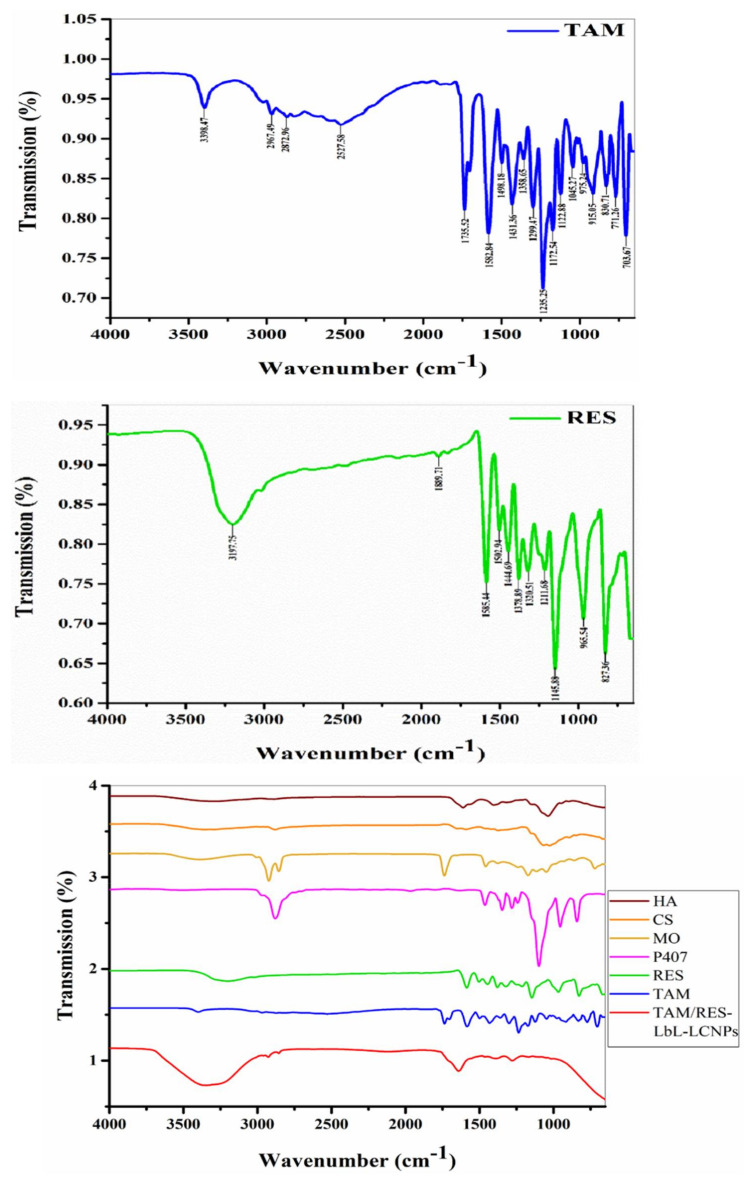
Fourier transform infrared spectroscopy analysis of TAM, RES, and other components involved in TAM/RES–LbL-LCNPs preparation. HA: hyaluronic acid; CS: chitosan; MO: glyceryl monooleate RES: resveratrol; TAM: tamoxifen citrate.

FTIR analysis is one of the best methods to estimate the chemical stabilization of the encapsulated compounds within the NPs. It is also essential for functional group identification existing in the TAM, RES, and the mixture involving TAM/RES–LbL-LCNPs and the auxiliary materials involved in the interaction: P407 and MO as a first step, and CS and HA as a second step (Figure 5).

The FTIR spectra of TAM, RES and TAM/RES–LbL-LCNPs were recorded in the spectral region (700–3500) cm^−1^. The FTIR spectra of the TAM absorption band at 3398.47 cm^−1^ were due to broad O–H stretching vibrations, while the bands at 2967.49 cm^−1^, 2872.96 cm^−1^, and 2527.58 cm^−1^ were because of aromatic C–H stretching. Additionally, the bands at 1735.52 cm^−1^, 1582.84 cm^−1^, and 1498.18 cm^−1^ were because of carboxylic C=O, aromatic C–C, and C=C ring stretching, respectively. Absorptions at 1299.47 cm^−1^, 1235.25 cm^−1^, and 1122.88 cm^−1^ were because of aromatic C–O stretching [55].

While FTIR spectra for the RES absorption band at 3197.75 cm^−1^ were due to O–H stretching, the bands at 1585.44 cm^−1^ and 1502.94 cm^−1^ were because of aromatic C–C and C=C ring stretching, respectively. The bands at 1211.63 cm^−1^ and 1145.88 cm^−1^ were because of aromatic C–O stretching [56]. Through the interaction of TAM and RES and the availability of auxiliary materials, the FTIR analysis revealed that they shared similar bands, which were O–H stretching (2500–3400) cm^−1^ and C–O stretching (1310–1380) cm^−1^. The result was (TAM/RES–LbL-LCNPs), and while examining FTIR, the following bands were obtained: The band at 3355.76 cm^−1^ was caused by O–H stretching. The bands at 2926.10 cm^−1^ and 2854.93 cm^−1^ were caused by aromatic C–H stretching. The bands at 1639.83 cm^−1^ and 1461.14 cm^−1^ were because of carboxylic C=O and C=C stretching, respectively. The bands 1244.23 cm^−1^ and 1140.90 cm^−1^ were caused by aromatic C–O stretching.

After all the results obtained and comparing the packages, we found that the final material (resulting) was shifting in O–H and C=O bands due to hydrogen bonding, which changed the shape and intensity of the absorption band, and it became more intense and broader on hydrogen bonding. Despite the shifting, the resulting functional group was the same as the functional group involved in the interaction. Consequently, these results confirmed the successful relationship between stilbenoid and polymer via intermolecular H-bonding. The wide –C=O stretching band (1759.14 cm^−1^) was caused by the polymer’s carbonyl and carboxyl group interactions, confirming the drug’s stable nature in the drug–polymer mixture [57].

### 3.6. Measurement of Particle Size and Morphology

**Figure 6:**
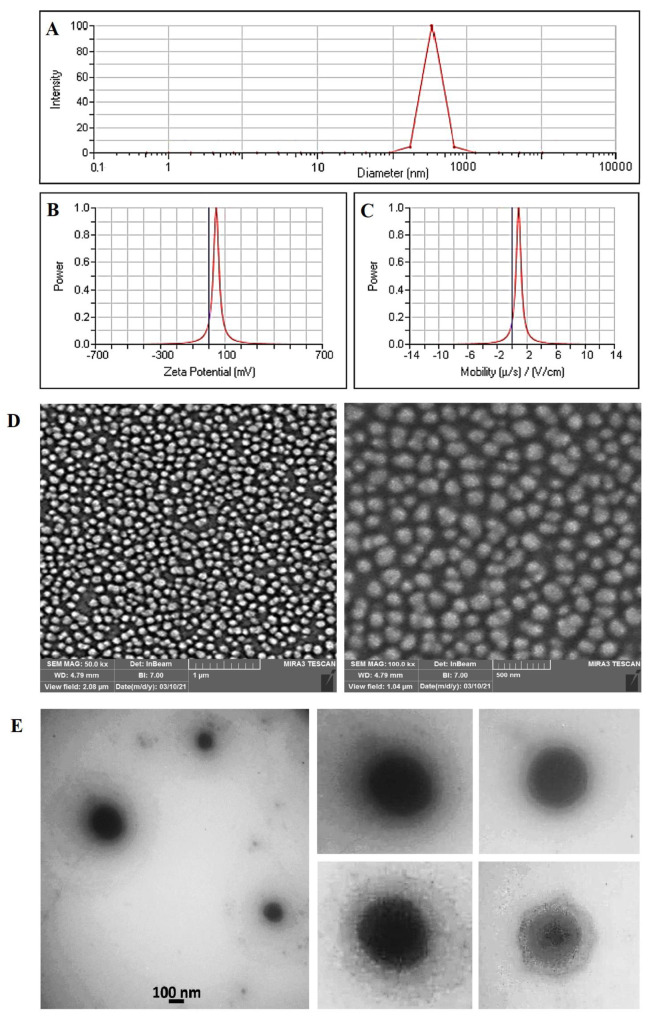
Physical characterization of TAM/RES–LbL-LCNPs: (**A**) Dynamic Light Scattering (DLS) analysis, (**B**) zeta potential analysis, (**C**) mobility analysis, (**D**) FE-SEM with a scale bar of 1 µm and 500 nm, respectively, and (**E**) TEM images with selected particles of synthesized TAM/RES–LbL-LCNPs (as presented in the right side).

Figure 6A shows the results of Dynamic Light Scattering (DLS) analysis for the synthesized TAM/RES–LbL-LCNPs. In this figure, the particle size of TAM/RES–LbL-LCNPs was shown to be about 217 nm, with a PDI value 0.089, confirming the formation of nanoparticles in the mono-distribution phase. To further characterize the TAM/RES–LbL-LCNPs, the zeta potential (ZP) reading was +45.05 mV, and mobility was 0.89 (Figure 6B,C), suggesting TAM/RES–LbL-LCNPs were formulated stable, as described by the guideline. The higher positive value can be explained by the presence of cationic CS in the TAM/RES–LbL-LCNP preparation. Zeta potential is the electrostatic potential in the particles’ shear plane, indicating the degree of attraction or repulsion between adjacent and similarly charged particles [47]. Generally, 20 mV, 30 mV, and 60 mV potential values indicates short-term, good, and excellent stabilities, respectively [58], whereas the −5 mV to 5 mV range refers to fast aggregation. The particle’s surface charge and drug’s binding ability with NPs critically define the drug desorption rate in the NP and loading efficiency. Whether on the center or surface of the NPs, encapsulated actively charged material location can be determined by the ZP value [59].

The spherical shape of TAM/RES–LbL-LCNPs was confirmed using SEM analysis. According to SEM images, synthesized TAM/RES–LbL-LCNPs are spherical, smooth, and almost homogenous structures of 200–205 nm (Figure 6D). The morphology of the prepared TAM/RES–LbL-LCNPs was characterized using TEM. As shown in Figure 6E, the magnified TEM image showed spherical particles and an outer layer around TAM/RES–LbL-LCNPs with an average particle size of 150–180 nm, which is slightly smaller than those obtained by DLS measurements. This observation may be due to the shrinkage of nanoparticles due to dehydration performed during TEM processing [60].

NP toxicity depends on various components, such as size, surface charge, composition, contamination, and specific surface area [61]. However, NP size critically influences cell uptake, organelle interaction, death mechanism, and clearance. NPs (size 10 to 200 nm) demonstrated increased permeability and amassed retention, specifically in cancer and not healthy cells [62].

### 3.7. In Vitro Hemolytic Activity

**Figure 7:**
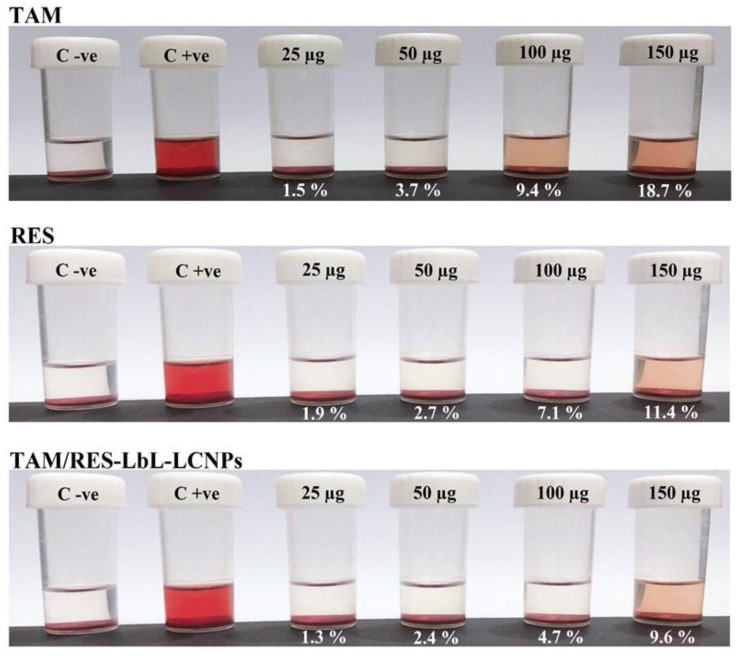
Hemolysis and blood smear stability of human RBCs. Percentage of hemolysis of TAM, RES, and other components involved in TAM/RES–LbL-LCNPs at different concentrations (25, 50, 100, and 150 µg mL^−1^) after 1-h incubation period at 37 °C.

**Figure 8:**
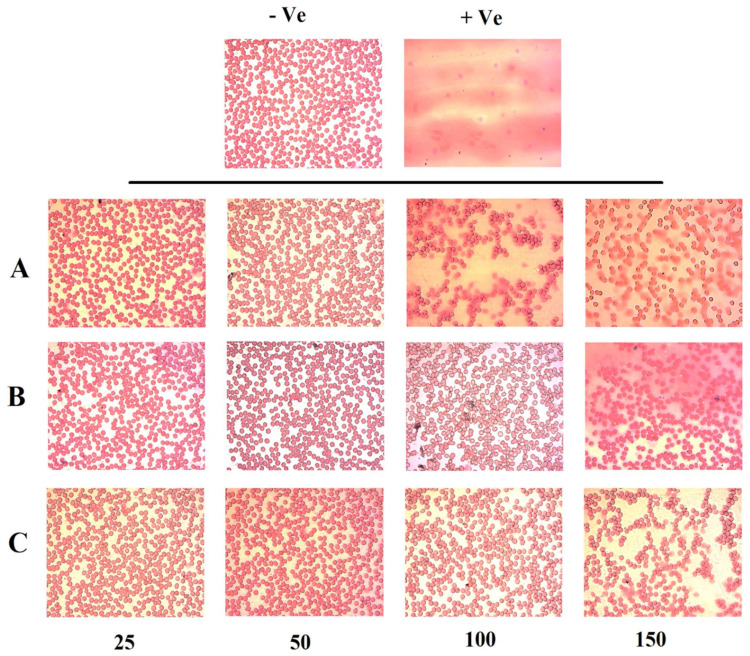
Microscopic images of human RBCs at the same conditions and concentrations: (**A**) TAM, (**B**) RES, (**C**) TAM/RES–LbL-LCNPs. Magnifications is 400X.

**Figure 9:**
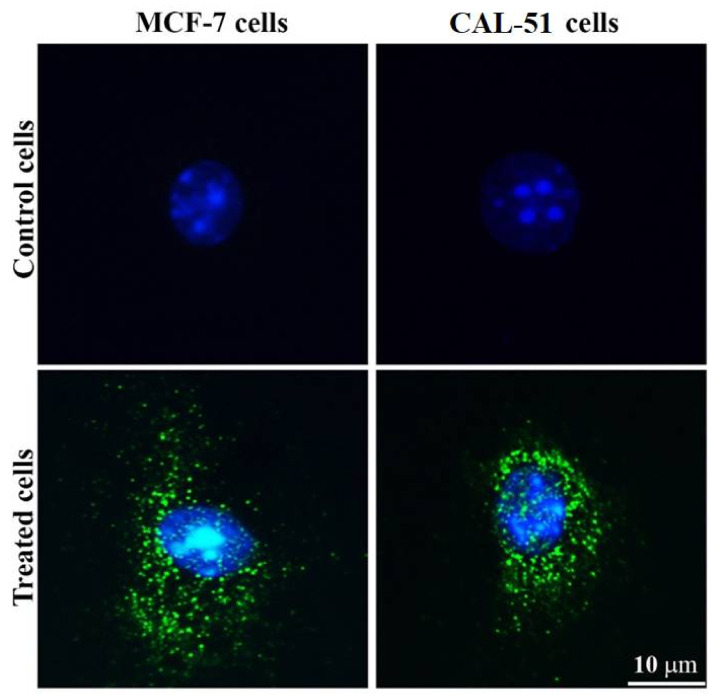
Uptake of fluorescent dye visualized by fluorescent microscopy. The cells were incubated with FITC-labeled TAM/RES–LbL-LCNPs for 24 h. Images were acquired at 400× magnification.

**Figure 10:**
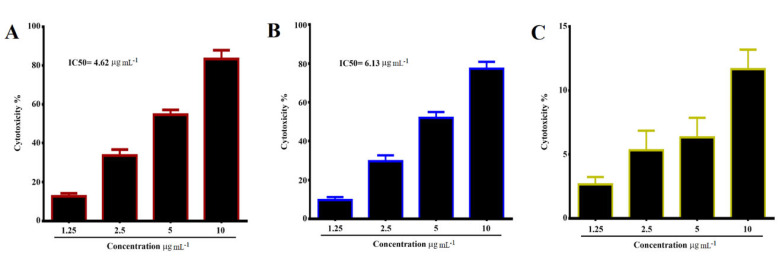
Growth inhibition of (**A**) MCF-7, (**B**) CAL-51 human breast cancer cell lines treated with different concentrations (1.25, 2.5, 5, and 10 μg mL^−1^) of TAM/RES–LbL-LCNPs. IC_50_ value for MCF-7 was 6.13 μg mL^−1^ and CAL-51was 4.62 μg mL^−1^, and (**C**) WRL-68 normal human liver cell line.

**Figure 11:**
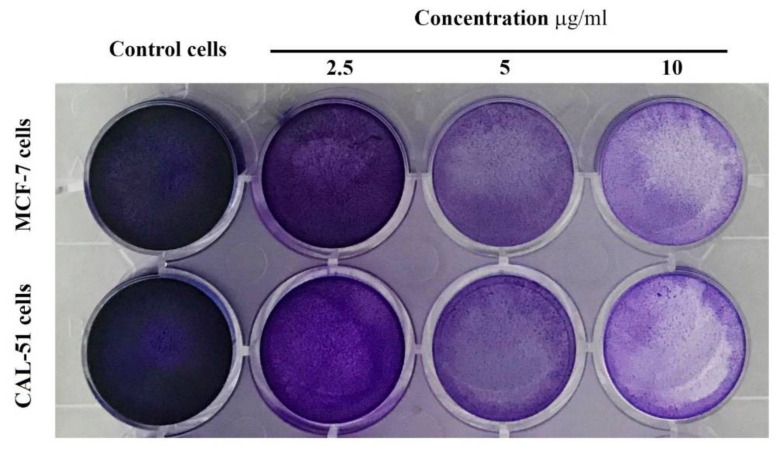
Colony formation assay of TAM/RES–LbL-LCNPs nanoparticles in cancer cell lines. MCF-7 cell line (upper line) and CAL-51 cell line (lower lane).

**Figure 12:**
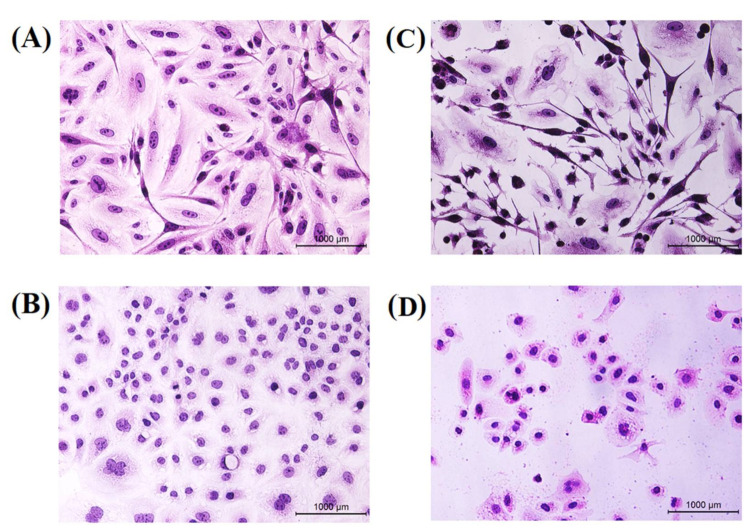
Morphological changes of hematoxylin and eosin. MCF-7 cell line (upper lane) and CAL-51 cell line (lower lane) were treated with IC_50_ concentration of TAM/RES–LbL-LCNPs for 24 h. (**A**,**B**) Non-treated cells show normal structure without prominent apoptosis at MCF-7. CAL-51, respectively (**C**,**D**) Cells treated with TAM/RES–LbL-LCNPs; apoptotic cells show condensed, and fragmented nuclei were recorded at MCF-7. CAL-51, respectively.

Low or negligible hemolytic activity is a pre-requisite in parenteral administration. Therefore, a blood interaction study was performed on all formulations encompassing TAM, RES, and TAM/RES–LbL-LCNPs. The leakage of hemoglobin from Red Blood Cells (RBCs) was used to compare the membrane-damaging properties of MO-based LCNPs quantitatively. Figure 7 represents the images of hemolysis activity of RBCs after treatment with four concentrations of TAM, RES, and TAM/RES–LbL-LCNPs (25, 50, 100, and 150 µg mL^−1^) along with normal saline and distilled water as negative and positive controls, respectively. It was observed that TAM/RES–LbL-LCNPs have fewer effects on RBCs than pure TAM and RES. The percentage of hemolysis of TAM/RES–LbL-LCNPs at the concentration 25 to 100 µg mL^−1^ was within the permissible level (1.3, 2.4, and 4.7%, respectively) of <5% hemolysis, while 150 µg mL^−1^ exceeded this level and was 9.6%. In contrast, the last two concentrations of pure TAM and RES exceeded the permissible level (9.4%, 18.7%, 7.1%, and 11.4%, respectively); however, 25 and 50 µg mL^−1^ were in the permissible range of <5% hemolysis (1.5%, 3.7%, 1.9%, and 2.7%) (Figure 4, Figure 5, Figure 6, Figure 7, Figure 8, Figure 9, Figure 10, Figure 11 and Figure 12). Erythrocytes are exceedingly prone to oxidative damage due to membranes’ elevated polyunsaturated fatty acid content and increased cellular oxygen and hemoglobin concentrations [63]. The results demonstrated the ability of TAM and RES to protect human RBCs against hemolytic damage and reduce oxidative stress under in vitro environments [64,65] attributed to membrane integrity preservation, modifications in cell membrane proteins and lipids, or cell membrane water content. The ability of RES to incorporate inside cell membranes is essential for cell protection efficiency by polyphenol compounds [66].

The hemolysis assay photographs demonstrated no harmful effect on RBC morphology from 25 to 150 µg mL^−1^ concentration, especially at TAM/RES–LbL-LCNPs. A light microscope image of RBCs (Figure 8) demonstrates the effect of TAM, RES, and TAM/RES–LbL-LCNPs at different concentrations, as observed in Figure 4, Figure 5, Figure 6, Figure 7, Figure 8, Figure 9, Figure 10, Figure 11 and Figure 12. TAM and RES showed RBC cell membrane destruction at high concentrations. It was clear that the hemolysis observed in RBCs treated with TAM was slightly higher than RES. Interestingly, TAM/RES–LbL-LCNPs demonstrated significantly decreased hemolysis due to the HA layer forming a protective cover that hindered the interactions between MO and RBCs and made the system more hemocompatible and safe.

Additionally, the ability of the HA coating to enhance the biocompatibility of the nanomaterials was previously reported [67]. Again, these results and concentrations of 25 to 100 µg mL^−1^ confirmed that successful LbL assembly of polyelectrolyte multilayer (PEM) on TAM/RES-LCNPs could be applied systemically, owing to <5% hemolysis. In accordance with the American Society for Testing and Materials (ASTM) E2524-08 standard (Test Method for Analysis of Hemolytic Properties of Nanoparticles), the hemolysis percentage that exceeds 5% elucidates RBC deterioration due to the tested samples [68].

### 3.8. Measurement of Cellular Uptake Activity

The conjugation of FITC to TAM/RES–LbL-LCNPs and their cellular uptake were observed under a fluorescent microscope (Figure 9). 

The treated MCF-7 and CAL-51 displayed increased intensity, confirming specific uptake of the compounds in the cell’s cytoplasm. Conversely, TAM/RES–LbL-LCNPs targeted various living cell organelles, demonstrating enhanced intensity owing to their smaller size. Moreover, the electrostatic interaction between the electrically charged cell membrane and the charged NPs may provide enhanced interaction and internalization rates [69]. The data demonstrate enhanced anti-cancer of TAM/RES–LbL-LCNPs in addition to apoptosis induction, membrane-penetrating capacity, biocompatibility, simple modification, and chemical synthesis. Furthermore, TAM/RES–LbL-LCNPs could make their way to the nucleus [70].

### 3.9. Cytotoxicity Using MTT Assay

Figure 10 demonstrates cytotoxicity data analyzed of MCF-7, CAL-51, and WRL-68 cell lines after 24 h of exposure with different concentrations of TAM/RES–LbL-LCNPs.

Treatment of the MCF-7 cell line with TAM/RES–LbL-LCNPs demonstrated concentration-dependent significant cell growth inhibition (*p* ≤ 0.05). However, after 24 h of treatment with 10 μg mL^−1^, the inhibition rate exceeded 79.6%, and the IC_50_ value was 6.13 μg mL^−1^. For the CAL-51 cell line, the same picture was drawn after treatment with TAM/RES–LbL-LCNPs, which showed concentration- and time-dependent significant cell growth inhibition. The present study proved that TAM/RES–LbL-LCNPs induced death of triple-negative (ER–/PR–/HER2–) and hormone receptor-positive (HER2+) breast cancer cells, and the CAL-51 cells were more sensitive to TAM/RES–LbL-LCNPs when compared with MCF-7 cells. Due to the antitumor effect of TAM and RES on breast cancer cells, it has been stated that TAM/RES–LbL-LCNPs can decrease cell viability and may induce apoptosis in treated cells leading the cells to remain in the G0/G1 phase while significantly reducing the cell proportion in the G2 phase and decreasing the S phase subpopulation [71]. The superior cytotoxicity of TAM/RES-NPs could be ascribed to the enhanced solubility of TAM/RES entrapped in LCNP formulations, leading to an increased concentration in the cell’s vicinity, thereby causing higher toxicity [32]. In addition, the rapid internalization of LCNPs into cells through endocytosis or phagocytosis led to constant and sustained exposure of cancer cells to the drug. In contrast, the free drug cellular uptake primarily relies on diffusion, which after a certain limit, decreased as a result of intracellular saturation, which prevented further drug internalization. Additionally, the specific interactions between the bio-adhesive lipophilic MO and cell membranes influenced the physicochemical properties of lipid bilayers and further facilitated LCNPs’ internalization.

### 3.10. Clonogenicity Assay

A clonogenic survival assay for MCF-7 and CAL-51 cells determined the long-term toxicity of TAM/RES–LbL-LCNPs at three concentrations. As shown in Figure 11, treatment with TAM/RES–LbL-LCNPs demonstrated a concentration-dependent decrease in colony numbers in both cancer lines compared to the non-treated cells. 

At various concentrations, the inhibitory efficacy of TAM/RES–LbL-LCNPs was less effective in MCF-7 than CAL-51 cells. The TAM/RES–LbL-LCNPs may stimulate many morphological changes in the cells that formed clusters with lower numbers of extensions and impaired cell–cell communication, while control cells did not demonstrate such alterations. Therefore, data from previous studies show that flavonoids and stilbenoids induce cell death *via* apoptosis or necrosis, inhibiting the survival of MCF-7, MDA-MB-231, and MCF10A cells, implying that resveratrol has anti-proliferative effects. Flavonoids and stilbenoid are two compounds with anti-cancer effects that can induce apoptosis in cancer cell lines [72].

### 3.11. Hematoxylin and Eosin Staining

Hematoxylin and eosin stains have been used for further confirmation to recognize the morphologic changes in treated cells with the synthesized TAM/RES–LbL-LCNPs. As seen in Figure 12, MCF-7 and CAL-51 cells revealed remarkable morphological changes in MCF-7 and CAL-51 cells and were characterized by cell swelling and cytoplasmic vacuolization and suffered from chromatin condensation starting from the periphery of the nuclear membrane leading to nuclear condensation. 

These are a common feature of apoptosis during late stages, and the condensed nucleus finally breaks up inside an intact cell membrane when equated to non-treated cells (Figure 12). Therefore, the present findings found that TAM/RES–LbL-LCNPs induced cell death in MCF-7 and CAL-51 cells via apoptosis. Therefore, a combinatory treatment with natural products such as flavonoids, alkaloids, polyphenols, and targeted therapy to augment the anti-cancer effect of conventional drugs represents a vital strategy to circumvent TNBC drug resistance against lines such as CAL-51 and MDA-MB-231 [73,74].

### 3.12. Acridine Orange-Ethidium Bromide Staining

**Figure 13:**
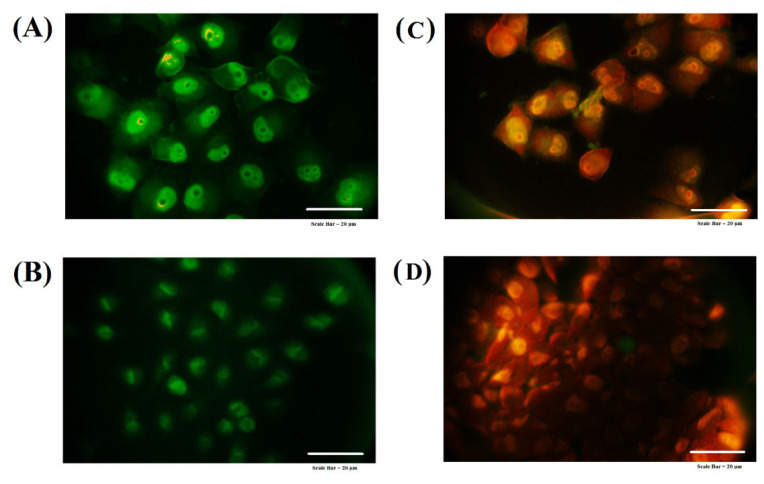
Apoptotic feature detected by acridine orange–ethidium bromide dual staining assay of the MCF-7 cell line (upper lane) and the CAL-51 cell line (lower lane). (**A**,**B**) Non-treated cells at MCF-7. CAL-51, respectively. (**C**,**D**) Cells treated with TAM/RES–LbL-LCNPs at MCF-7. CAL-51, respectively.

To further confirm the potential of TAM/RES–LbL-LCNPs in stimulating death in both cancer cell lines, AO/EtBr staining was carried out (Figure 13). 

MCF-7 and CAL-51 cells treated with TAM/RES–LbL-LCNPs showed aggressive membrane integrity loss compared to untreated cells showing nuclei with an intact structure and a stable, bright green color, whereas fluorescence and morphological tests recognized the significantly enhanced changes, such as chromatin condensation, in the treated cells. The apoptotic cell nucleus color varies from red to orange and chromatin with diverse condensation or fragmentation levels. However, the morphological modifications in cells treated with TAM/RES–LbL-LCNPs indicate apoptosis instead of necrosis-induced cell death. Previous studies reported [75,76] enhanced therapeutic effects by TAM on cancer cells (MCF-7, T47D, and MDA-MB231) through apoptosis and reducing the TAM toxicity in healthy cells [75]. TAM induces apoptosis and cell accumulation in the G0/G1 phase [76,77].

Moreover, the previous results indicated that resveratrol significantly decreased viability and induced apoptosis in human gastric adenocarcinoma cells (SGC-7901) cells in a dose- and time-dependent manner within a specific range [78]. The results demonstrated that resveratrol decreases cell viability by inducing apoptosis and G2/M-phase cell cycle arrest. Additionally, resveratrol inhibited 65% cell proliferation in MCF-7, indicating the antiproliferation activity and another experiment to determine apoptosis by AO/EtBr also showed cell death [79]. Overall, it suggests these compounds have shown anti-cancer activities and stimulate apoptosis.

### 3.13. Expression of p53 and Caspase-8

**Figure 14:**
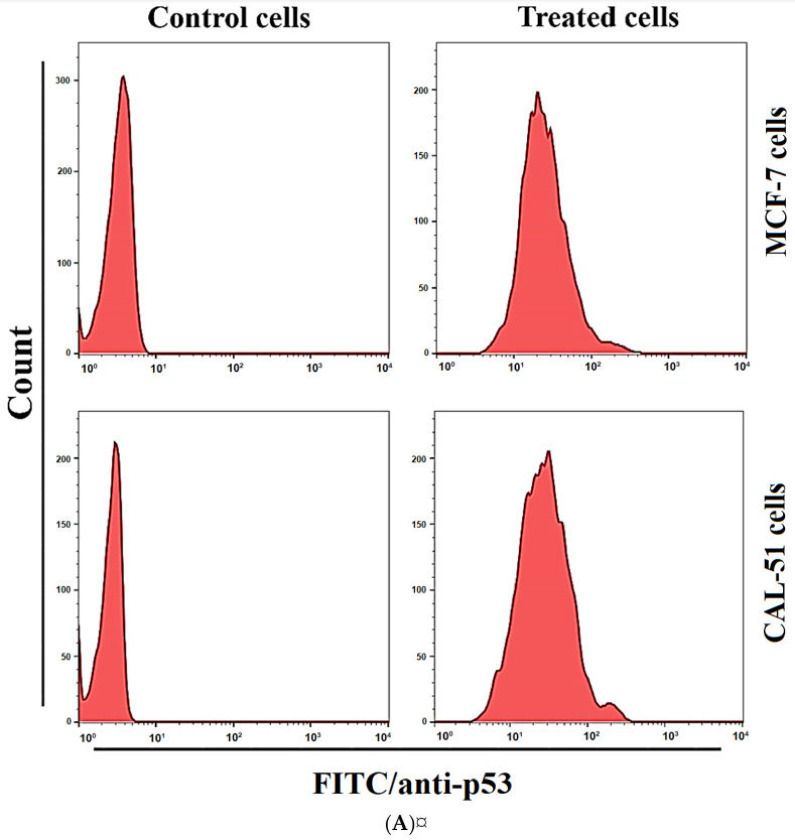

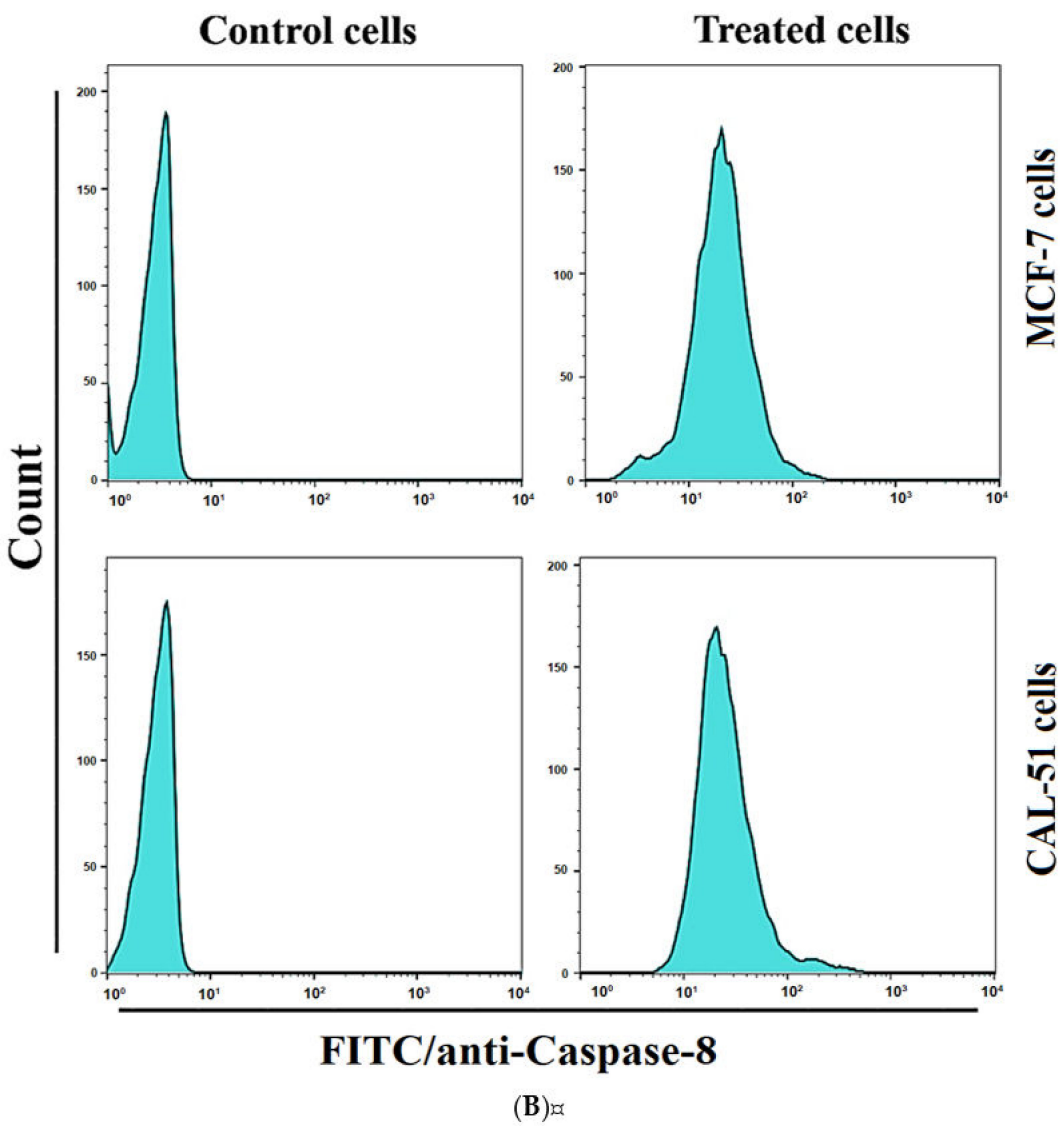
TAM/RES–LbL-LCNPs regulated p53 (**A**) and caspase-8 (**B**) levels in MCF-7 and CAL-51 cells. A flow cytometer assessed fluorescence histograms of p53 and caspase-8.

Expression of apoptosis markers, p53 and caspase-8, was investigated to elucidate cell proliferation inhibition mechanisms in the MCF-7 and CAL-51 cell lines. As shown in Figure 14, the treatment with TAM/RES–LbL-LCNPs at IC_50_ concentration for 24 h revealed that the protein levels for p53 and caspase-8 were different in MCF-7 and CAL-51 cell lines. 

For p53, the expression increased in treated MCF-7 and CAL-51 cell lines than non-related cell lines. These results also exhibited an increase in expressions of caspase-8 in treated MCF-7 and CAL-51 cell lines than non-related cell lines. Thus, these results showed that treatments by administering the TAM/RES–LbL-LCNPs induced apoptosis by increasing p53 and caspase-8, important modulators for the apoptotic pathway, in both the MCF-7 and CAL-51 cell lines.

Given that resveratrol has been proposed to have chemo-preventive and chemotherapeutic effects, the previous investigation evaluated the significance of resveratrol in overcoming tamoxifen resistance when evaluated against MCF-7 and MCF-7/TR cells through reduction of Akt and ERK1/2 phosphorylation and activation for caspase-3 and PARP cleavage [80]. The data is consistent with a previous study that demonstrated increased caspase-3 and -8 levels leading to resveratrol-induced apoptosis of SGC-7901 [78]. Furthermore, another study demonstrated that RES induced apoptosis of human breast cancer cells in ER-negative and ER-positive cells [81]. Hence, our data offers a new and safer way to promote the mechanisms using phytochemicals in combinatorial therapy.

### 3.14. In Vivo Toxicity Assay

**Figure 15:**
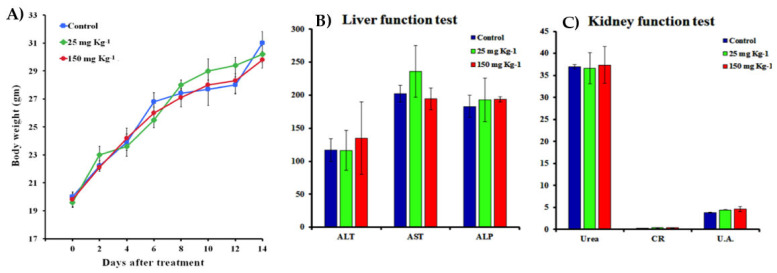
(**A**) Body weight changes for mice treated with TAM/RES–LbL-LCNPs at doses of 25 and 150 mg kg^−1^ after 14 days. (**B**,**C**) Mice treated with TAM/RES–LbL-LCNPs were sacrificed, and blood was extracted and analyzed for biochemical parameters in the serum for liver and kidney functions. Non-treated animals were used, and non-significant changes were recorded. Data were analyzed by the differences between the doses and the control group. Alanine transaminase (ALT), aspartate transaminase (AST), alkaline phosphatase (ALP), creatinine (CR), and uric acid (UA).

Based on the *in vitro* evaluations, the *in vivo* experiments were further investigated to determine the toxicity of TAM/RES–LbL-LCNPs using the mice model (Figure 15). 

Administration with TAM/RES–LbL-LCNPs in the dose range of 25 to 150 mg kg^−1^ for 7 or 14 days caused no mortality, with no difference in body weight between the TAM/RES–LbL-LCNP-treated and non-treated mice (control). Taken together, TAM/RES–LbL-LCNPs treatment did not cause any apparent toxicity or visible organ damage in TAM/RES–LbL-LCNPs group. Our data is consistent with previous studies involving TAM combined with natural products [82,83].

It is necessary to investigate whether TAM/RES–LbL-LCNPs cause toxicity in the liver and kidney. Thus, the present investigation determined levels of alanine transaminase (ALT), aspartate transaminase (AST), blood urea nitrogen, and creatinine (Figure 15). Urea and creatinine are metabolites associated with kidney functionality. The ALT, AST, and ALP levels in blood tested as a hepatic functionality indicator were not significantly different (*p* ≥ 0.05). In addition, the levels of uric acid were also determined with a non-significant decrease (*p* ≥ 0.05).

**Figure 16:**
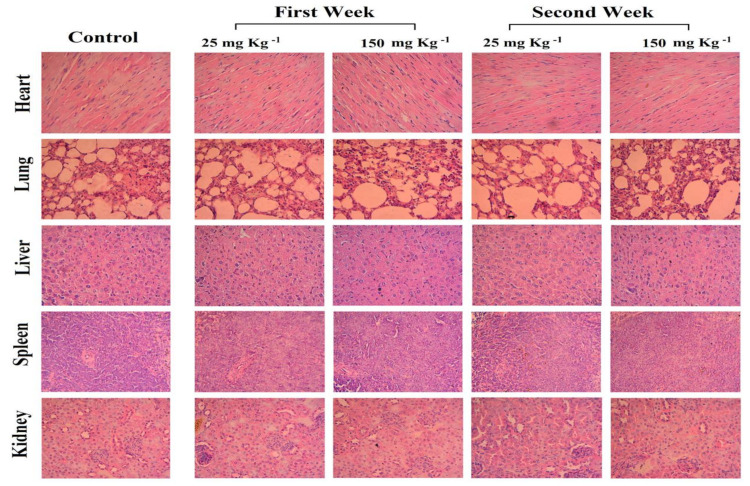
Photomicrographs of histological assessment of various mice organs after 7 and 14 days of inoculation intraperitoneally with TAM/RES–LbL-LCNPs at doses of 25 and 150 mg kg^−1^. The liver, kidneys, heart, lung, and spleen were removed, and tissue sections were subjected to H&E staining, followed by a histopathological evaluation by light microscopy. Magnifications is 400X.

Furthermore, the histopathological examination of tissues was performed to determine whether two doses of TAM/RES–LbL-LCNPs (25 and 150 mg kg^−1^) could cause tissue damage, inflammation or lesions in the liver, kidneys, heart, lung, and spleen (Figure 16). No apparent damages or histopathological abnormalities were observed for treated mice. The treatment with TAM/RES–LbL-LCNPs showed organ architecture almost similar to the control and did not display any toxicity, thereby providing an opportunity further to assess the role of TAM/RES–LbL-LCNPs in *in vivo* models.

Jian et al. showed that TAM-PLGA (poly D, L-lactic-co-glycolic acid)-NPs significantly reduced the hepatotoxicity, while co-encapsulation of QT (quercetin) and TAM in PLGA-NPs fully eliminated TAM induced hepatotoxicity [84]. Furthermore, when combined with TAM and loaded into nanostructured lipid carriers, sulforaphane significantly reduced the toxicity associated with TAM [82]. It was recorded that RES could synergize with TAM and trigger apoptosis in MCF-7/TR cells [80]. Therefore, the present strategy might be vital for developing safe cancer chemotherapeutics delivery and an apoptotic inducer in breast cancer cells.

## 4. Conclusions

The TAM/RES–LbL-LCNPs were prepared by the hydrotrope method, coated with multiple layers of positively charged CS and negatively charged HA, and many characterization methods were performed. The findings revealed that TAM/RES–LbL-LCNPs were therapeutically efficient, safe, and biocompatible for human red blood cells with no noticeable toxicity, adverse side effects, or behavioral abnormalities in mice implying their safety in biomedical applications. Furthermore, the study also proved that the TAM/RES–LbL-LCNPs possessed potent cytotoxicity and could enhance apoptosis in both types of human breast cancer cell lines, MCF-7 and CAL-51, with less toxicity against WRL-68. These potentials were anticipated due to the effectiveness of TAM/RES–LbL-LCNPs on the cell nucleus and its induction of DNA damage, which, when left unrepaired, through non-treatment, caused cell death. Moreover, the ability of TAM/RES–LbL-LCNPs to degrade DNA and be toxic to the cancer cells makes it useful in developing a safe cancer chemotherapeutics delivery system and apoptotic inducer in breast cancer cells. Hence, our findings provide a new and safer way of using phytochemicals in combinatorial therapy.

## Data Availability

Not applicable.

## Supplementary Materials

The following are available online at https://www.mdpi.com/article/10.3390/pharmaceutics13071098/s1, Figure S1: Image showing i.p. injection of TAM/RES–LbL-LCNPs. Figure S2: Image showing the dissection of mice at the end of the experiment. Figure S3: Image showing isolated mouse organs kidney, liver, spleen, heart, and lungs at the end of the experiment.
