# Supplementary material for: Layer-by-Layer Nanoparticles of Tamoxifen and Resveratrol for Dual Drug Delivery System and Potential Triple-Negative Breast Cancer Treatment
Source: Pharmaceutics. 2021 Jul 20;13(7):1098. doi: 10.3390/pharmaceutics13071098 (PMC8309206; doi:10.3390/pharmaceutics13071098)
Supplement: Supplementary file 1 [file pharmaceutics-13-01098-s001.zip › pharmaceutics-1286364-supplementary.pdf]

pharmaceutics

MDPI

Supplementary Materials: Layer-by-Layer Nanoparticles of Tamoxifen and Resveratrol for Dual Drug Delivery System and Potential Triple-Negative Breast Cancer Treatment

Ali A. Al-jubori, Ghassan M. Sulaiman, Amer T. Tawfeeq, Hamdoon A. Mohammed, Riaz A. Khan and Salman A. A. Mohammed

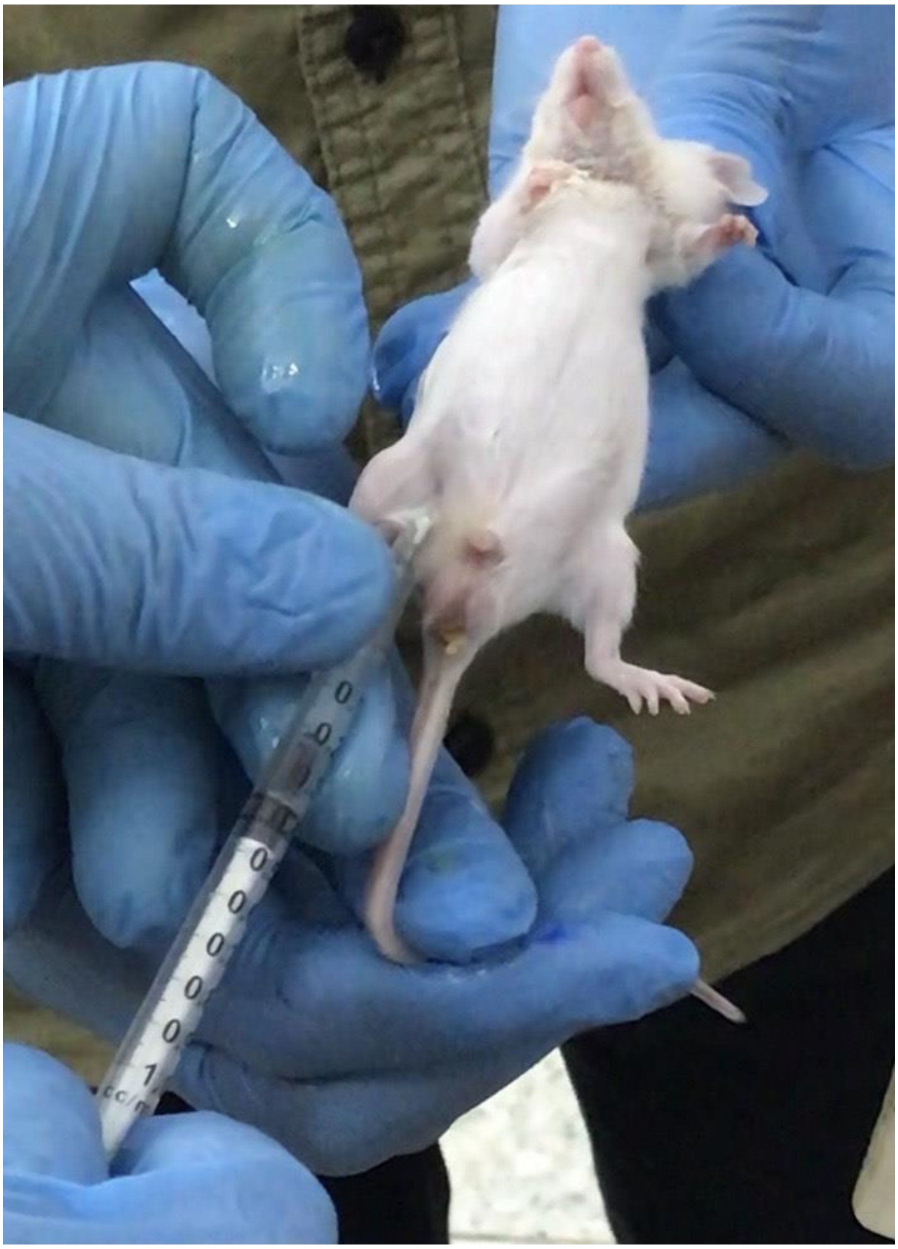

Figure S1. Image showing intraperitoneal (i.p.) injection of TAM/RES-LbL-LCNPs.

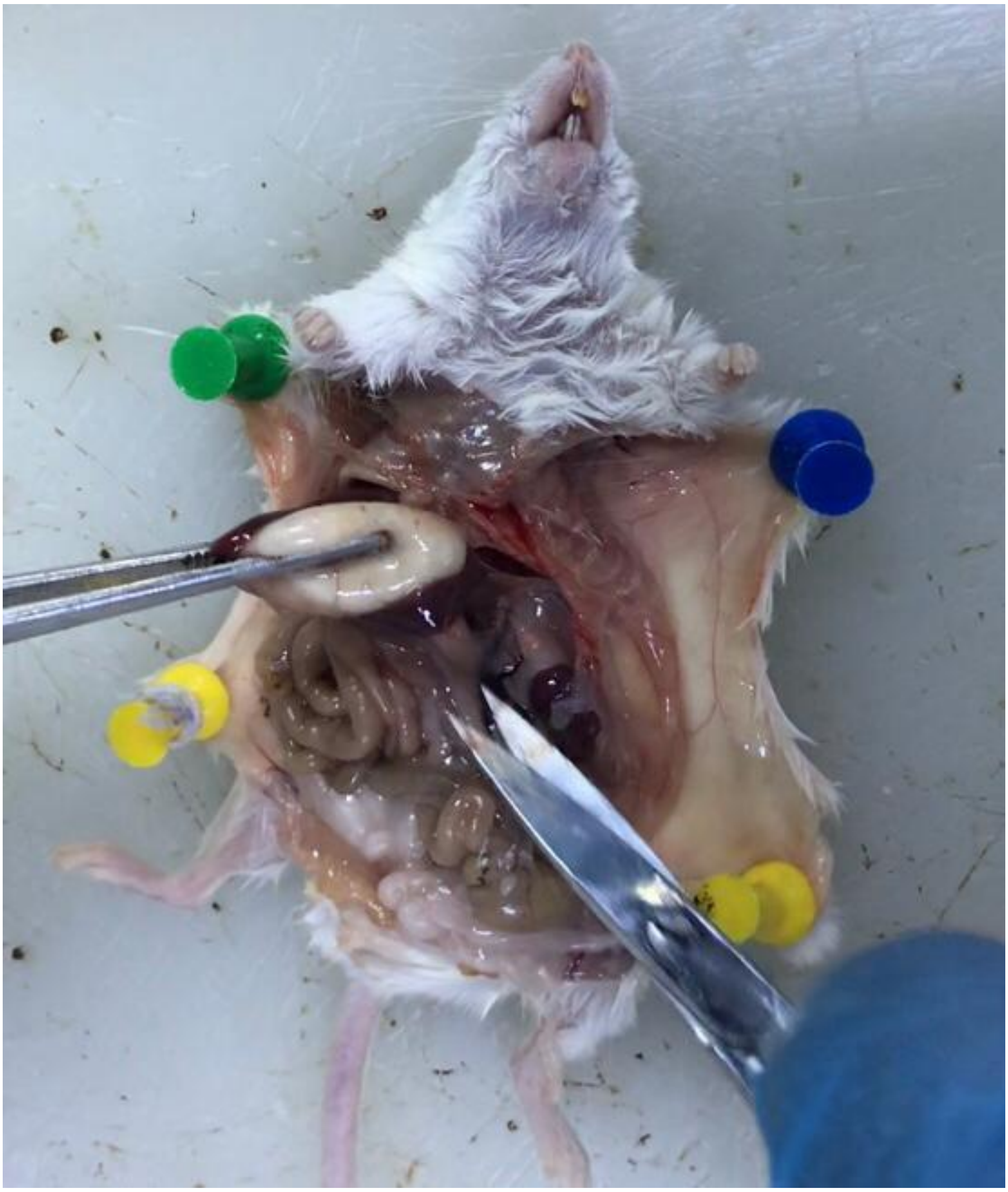

Figure S2. Image showing the dissection of mice at the end of the experiment.

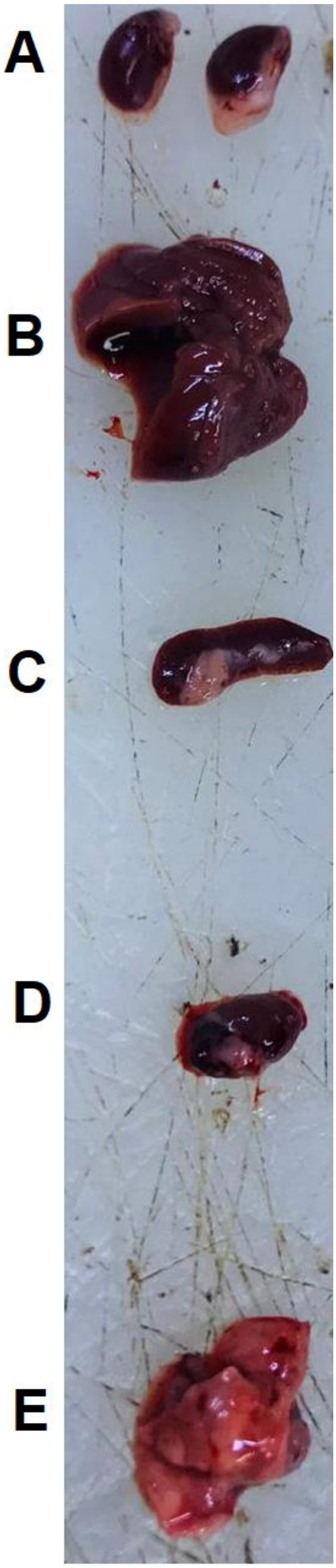

Figure S3. Image showing isolated mouse organs kidney (A), liver (B), spleen (C), heart (D) and lungs (E) at the end of the experiment.
